# OTME-4. IDH mutated gliomas promote epileptogenesis via D-2-hydroxyglutarate dependent mTOR hyperactivation

**DOI:** 10.1093/noajnl/vdab070.055

**Published:** 2021-07-05

**Authors:** Armin Mortazavi, Islam Fayed, Muzna Bachani, Dragan Maric, Tyrone Dowdy, Mioara Larion, Alexander Ksendzovsky, Kareem Zaghloul

**Affiliations:** 1 National Institute of Health, Bethesda, MD, USA; 2 Medstar Georgetown University Hospital, Washington, DC, USA; 3 University of Maryland, Baltimore, MD, USA

## Abstract

Epilepsy in the context of brain tumors provides a great burden in these patients, yet mechanisms underlying this process are poorly understood. It has been demonstrated that isocitrate dehydrogenase (IDH) mutations are an independent factor in epileptogenesis in patients with low grade gliomas. Here, using electrographically sorted human cortical tissue from patients with IDH mutated tumor related epilepsy and *in vitro* cortical cultures, we explore a metabolic paradigm and its impact on increased neuronal excitability. We hypothesize the IDH mutation promotes epileptogenesis through its neomorphic activity of D-2-hydroxyglutarate (D-2-HG) production in turn interrupts surrounding normal neuronal circuitry potentially through metabolic perturbations. We demonstrate D-2-HG increases neuronal spiking activity, promotes distinct metabolic profiles independent of neuronal spiking activity, as well as increases neuronal mTOR signaling, which is reflected in human peritumoral epileptic cortex. Increased mTOR signaling is sufficient to upregulate neuronal spiking activity and, reciprocally, inhibition of mTOR corrects neuronal activity as well as partially corrects metabolic reprogramming. Our results suggest D-2-HG can lead to mTOR activation within the peritumoral neurons, thereby suggesting an additional possible mechanism of epileptogenesis in patients with IDH mutated low grade gliomas. Ultimately, our results raise the possibility of mTOR inhibition may be a promising treatment of seizures in patients with these tumors.

